# Frequency of Clinical Symptoms Associated With Incidental Paranasal Sinus Anomalies on MRI Brain in Patients Reporting to a Tertiary Healthcare Centre in Pakistan

**DOI:** 10.7759/cureus.109059

**Published:** 2026-05-17

**Authors:** Umair Ajmal, Shahabuddin Siddiqui, Ayesha Munir, Aamena Irfan Shami, Mohamed A Omran, Ahmed Eldeeb, Muhammad A, Ambreen Farooq, Maliha Baseer

**Affiliations:** 1 Radiology, Advanced International Hospital, Islamabad, PAK; 2 General Surgery, University Hospitals Bristol and Weston NHS Foundation Trust, Weston-super-Mare, GBR; 3 Vascular Surgery, East Suffolk and North Essex Trust NHS Foundation Trust, Colchester, GBR; 4 Medicine and Surgery, Advanced International Hospital, Islamabad, PAK

**Keywords:** abnormalities of paranasal sinuses, brain magnetic resonance imaging, incidental mri finding, maxillary sinus mucosal thickening, mucosal thickening, patient symptoms, polypoidal mucosal thickening

## Abstract

Background: Magnetic resonance imaging (MRI) of the brain is an increasingly advised investigation in clinical settings for various indications. There is a concurrent and proportionate rise in the incidence of various paranasal abnormalities, reported on this modality.

Objective: The objective of this study was to report the frequency of clinical symptoms in incidental paranasal sinus anomalies in patients undergoing MRI of the brain in a tertiary care hospital.

Methodology: This prospective observational study was conducted at a tertiary healthcare setup in Pakistan from January 16 to July 16, 2024, with a total of 214 individuals who underwent brain MRI. The images were examined for the presence of any incidental paranasal sinus abnormalities. Any symptomologies reported by respective individuals having these structural anomalies on MRI brain were then recorded.

Results: Of the total 214 patients included in the study, 61.2% (n=131) were male, with a mean age of 47.47±10.60 years, and 38.8% (n=83) were female, with a mean age of 48.08±10.16 years. Of the 214 patients, 35.5% (n=76) were found to have one incidental finding on their paranasal sinuses, while 12.6% patients (n=27) showed two or more incidental abnormalities. The remaining 111 patients (51.8%) had no paranasal sinus anomalies on their brain MRIs. Of the 103 patients with incidental paranasal sinus anomalies on MRI brain, 41% (n=43) had at least one symptom. The presence of mucosal thickening of >2mm and >4mm was more likely to be associated with symptoms like nasal blockage, headache, and allergic rhinitis, with a significant p-value of 0.04 and 0.02, respectively. Male patients are more likely to have symptomatic ethmoid sinus mucosal thickening than their female counterparts.

Conclusion: The paranasal sinuses anomalies may frequently be encountered on MRI brain imaging. The patients having a mucosal thickening of more than equal or more than 2 mm on MRI brain are more likely to have corresponding symptoms. Other structural abnormalities may not result in any significant symptoms.

## Introduction

Magnetic resonance imaging (MRI) of the brain is a frequently advised investigation in tertiary healthcare setups, both as an emergency and an elective diagnostic procedure to rule out intracranial illnesses. Since it includes the paranasal sinuses in a considerable number of cases, especially when ordered for symptoms like headache and vertigo, the incidental findings of this region are becoming a common phenomenon with increased frequency of MRI brain in radiological setups [1,2].

The nature of these incidental findings has been variable across the globe, as reported in the previous studies, ranging from thickening of the mucosa to retention cysts and paranasal polyps. There have been incidents where paranasal pathologies have been picked up by MRI performed for indications other than paranasal sinus pathologies [3-5]. Previous studies in western populations have reported a variable incidence of incidental paranasal sinus pathologies on brain MRI, ranging from 14% to 60%; data from developing countries are relatively sparse [4,5].

In busy tertiary healthcare centres where the patient burden is high, such incidental findings may create confusion and concerns for the patients, radiologists, and treating physicians alike, especially where history and symptomology related to paranasal sinuses are not mentioned on the MRI forms [1]. The aim of our study was to report the frequency of clinical symptoms in incidental paranasal sinus anomalies on MRI brain for diagnostic/therapeutic purposes in patients reporting to a tertiary healthcare setup in Pakistan. The study also corroborated the findings of previous studies on incidental findings in the paranasal sinuses.

## Materials and methods

This was a prospective observational cross-sectional study conducted at Advanced International Hospital, a tertiary healthcare hospital in Islamabad, Pakistan, from January 16 to July 16, 2024, involving a multi-disciplinary team from various hospital departments. The study was approved by the Ethical Committee of Advanced International Hospital (reference number: IRB#133-09/24).

Sample size

The sample size of the study was calculated using the WHO calculator with power of the study as 80%, confidence level at 95%, reference population proportion as 20% [5], and a two-tailed test; the minimum sample size turned out to be 194. After accounting for a 10% non-response/incomplete data rate, the final sample size turned out to be 214 patients.

Eligibility criteria

All the patients reporting to the radiology department for MRI brain for any indications and new signs and symptoms, other than paranasal sinus pathologies, were included in the study, while all those patients with a history of any surgery or radiation therapy, patients with a known/pre-existing diagnosis of chronic sinusitis, allergic rhinitis, or other sinonasal disorders were excluded from the study. Those unwilling to provide their history and data were also excluded from the study. The age, gender, drug history, any medical or surgical comorbidities, and other demographic details of the patients were noted.

Study procedure

The induction of the volunteers in the study was performed at the time of their reporting to the radiology department for their MRI brain imaging. Informed, explicit consent was obtained from all the patients before their induction into the study. A specially designed questionnaire was used to inquire about the presence of any symptoms related to paranasal sinuses (see Appendices). The patients were then classified as “symptomatic” if they had at least one symptom related to paranasal sinuses or “asymptomatic” if no symptom was reported.

The MRI brain imaging was performed via a 1.5 T MAGNETOM Sola MRI system (Siemens AG, Munich, Germany). The sequences used were T1, T2, fluid-attenuated inversion recovery (FLAIR), clinically isolated syndrome (CIS), susceptibility weighted imaging (SWI), diffusion-weighted imaging (DWI), and apparent diffusion coefficient (ADC). A slice thickness of 3 mm and a field view of 240 mm were used. Before performing the MRI, the patients were briefed in detail by a specially trained team of radiology technologists regarding the imaging procedure to allay their fears and concerns and to rule out any contraindications to MRI.

For all the patients, the paranasal sinus anomalies were recorded and classified according to their type and severity. Furthermore, the location of the anomalies (i.e., maxillary, ethmoid, frontal, or sphenoid), laterality, size, and the specific involved portion of the anatomical sites were also recoded. These were recognised as sinus mucosal thickening, sinus retention cysts, and sino-nasal polyps, etc. Further grading of paranasal thickening was done into three classes, i.e., ≤2 mm, 2.1-4 mm, and >4 mm, being the highest degree of mucosal thickening.

The data were entered and analysed using the IBM SPSS Statistics for Windows, version 25 (IBM Corp., Armonk, New York, United States). The quantitative variables were expressed in the form of mean and standard deviation (SD), while the qualitative variables were represented in the form of frequency and percentages. The Chi-square test was employed to evaluate further statistics, and a p-value of ≤0.05 was considered significant.

## Results

A total of 214 patients were included in the study. Of these, 61.2% (n=131) were male, with the mean age of 47.47±10.60 years, and 38.8% (n=83) were female, with the mean age of 48.08±10.16 years. The age of the patients in the cohort ranged from 18 to 66 years, with the mean age being 46±10.38 years. Around half of the patients (48.1%, n=103) were found to have incidental paranasal sinus anomalies on MRI brain; 76/214 (35.5%) had one incidental finding, while 27/214 (12.6%) patients showed two or more incidental abnormalities. The remaining 51.8% (n=111) of the patients studied had no paranasal sinus anomalies on their brain MRIs. These findings are summarised in Table 1.

**Table 1:** Demographic parameters and presence of incidental paransal sinus anomalies in patients ungergoing MRI brain. Data given as frequency (percentage) except in age, which is given as mean±SD NOTE: The percentages are based on the total number of anomalies identified, not the number of patients.

Parameter	Categories	Frequency (Percentage)
Gender	Male	131 (61.2%)
	Female	83 (38.8%)
Age (years), mean ± SD	Male	47.47±10.60
	Female	48.08±10.16
Presence of paranasal sinus anomalies on MRI Brain (n=214)	Yes	103 (48.1%)
	No	111 (51.8%)
Presence of symptoms in patients with paranasal sinus anomalies on MRI Brain (n=103)	No Symptoms	60 (58.2%)
	Symptoms Present	43 (41.7%)

**Figure 1:**
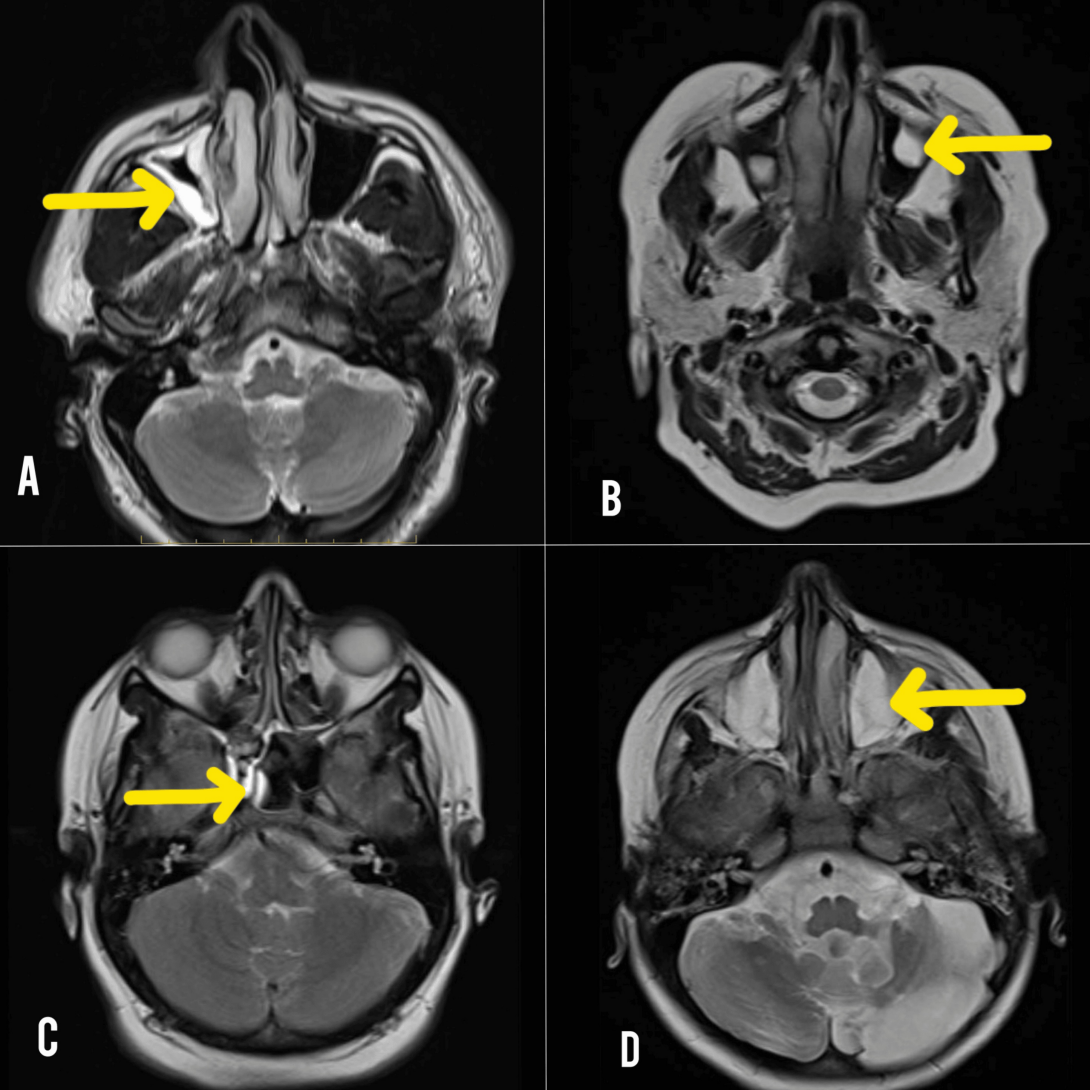
Representative images of different patients in the study with varius mucosal anomalies (A) Mucosal thickening >5 mm in the right maxillary sinus; (B) Mucous retention cysts in right and left maxillary sinuses; (C) Mucosal thickening >2 mm in the right sphenoid sinus; (D) Oppacification of bilateral maxillary sinuses

Representative images of mucosal anomalies are depicted in Figure 1.

As far as symptoms are concerned, of the total 103 patients with paranasal sinus MRI findings, around 41.7% (n=43) reported at least one symptom related to the paranasal sinus system. The remaining patients (58.2%; n=60) with incidental paranasal sinus anomalies on MRI brain reported no symptoms at all. The most commonly reported paranasal sinus system-related symptom amongst our cohort undergoing MRI brain was headache (83.4%, n=86), sensation of heaviness in the head (37.8%, n=39), post-nasal drip (34.9%, n=36), and facial pain (25.2%, n=26).

It should be noted that in the study cohort of 214 patients, headaches and seizures of all types were found to be the reason for performing MRI in 89% (n=190) of the patients. The most frequently encountered incidental paranasal sinus anomaly was mucosal thickening, seen in 29.9% (n=64/214). This was followed by air-fluid levels in 7.9% (n=17) and retention cysts in 7% (n=15) of the patients. Paranasal polyps were the least common anomaly found in 3.2% (n=7) of the patients. These findings are summarised in Table 2.

**Table 2:** Incidental mucosal anomalies and thier association with the symptoms in patients undergoing MRI brain

Paranasal sinus anomalies on MRI Brain	Incidence in total cohort (n=103/214)		Incidental anomalies with symptoms (n=43/103)	Incidental Anomalies with no symptoms (n=60/103)	p-value
Mucosal thickening		64 (62%)	38 (37%)	26 (25.5%)	0.06
Air-Fluid Levels		17 (16.5%)	3 (2.9%)	14 (13.5%)	0.07
Retention Cysts		15 (14.5%)	2 (1.9%)	13 (12.6%)	0.06
Paranasal Polyps		7 (6.7%)	Nil	7 (6.7%)	<0.001

In the 64 patients showing sinus mucosal thickening on MRI brain, classification of the mucosal thickening as per severity was done, which showed that 39% (n=25) had mucosal thickening of >4 mm, around 48.5% (n=31) had a thickening of 2-4 mm, while ≤2 mm of mucosal thickening was manifested in 12.5% (n=8). Here, the most symptomatic patients were those having a mucosal thickening of more than 4 mm (n=25), of which 68% (n=17) of the patients reported at least one symptom. This was followed by patients, with a mucosal thickening of 2-4 mm (n=31), of whom 58% (n=18) of patients had at least one symptom. These findings were statistically significant with a p-value of 0.02 and 0.04, respectively (Table 3).

**Table 3:** Distribution of patients with symptomatic and asymptomatic mucosal thickening as per severity

Severity of mucosal thickening	Incidence in patients showing paranasal findings on MRI (n=64/103)	Patients with symptomatic mucosal thickening (n=38/64)	Patients with asymptomatic mucosal thickening (n=26/64)	p-value
≤2 mm	8 (12.5%)	1 (2.6%)	7 (27%)	0.08
2-4 mm	31 (48.4%)	18 (47.4%)	7 (27%)	0.04
>4 mm	25 (39.1%)	19 (50.0%)	12 (46%)	0.02

In the 64 patients showing sinus mucosal thickening on MRI brain, the most common symptom in patients was headache (82.8%, n=53) (p-value 0.01) and heaviness (75%, n=48) (p-value 0.01), while post-nasal dribble was noted in 29.7% (n=19) (p-value 0.006). Of the patients with with mucosal thickening of ≤2 mm, only one (2.5%) reported symptoms of headache and left maxillary sinus pain, which was statistically insignificant (p-value=0.08). The most frequent mucosal thickening was seen in the ethmoid sinus (71.8%, n=46) (p-value=0.001) and maxillary sinus (65.6%, n=42) (p-value=0.003), while the least commonly involved sinus was the frontal sinus (14%, n=9) (p-value=0.04). Among patients with mucosal thickening in the ethmoid sinus (n=46), it was seen to be more common in male patients (58.6%, n=27 vs 41.3%, n=19, p-value 0.03). However, the same comparison was found statistically insignificant for other paranasal sinuses. With regard to sinuses showing mucosal thickening, the left ethmoid sinus showed more frequent mucosal thickening (n=32), followed by right maxillary sinus (n=19) and the right ethmoid sinus (n=14).

## Discussion

MRI brain is increasingly becoming a popular diagnostic tool for intracranial pathologies amongst clinicians. The investigation is favoured mainly because of its high sensitivity for detecting soft tissue pathologies with no radiation exposure as compared to other investigations like X-rays and computed tomography scans [3,6]. With easier availability in tertiary healthcare setups, MRI brain is frequently performed in clinical setups; consequently, an increase in the incidental findings becomes only natural [3,7].

Our study focused on the incidental findings of paranasal sinuses on MRI brain and sought to report the frequency of clinical symptoms reported with these findings, to determine the significance of these incidental findings. We found that 41% (n=43/103) of the patients with incidental paranasal sinus anomalies on MRI brain were found to have at least one symptom. We also found that the most common paranasal sinus abnormality on MRI brain was mucosal thickening, seen in around a third of the total cohort (29.9%, n=64/214), more than half of which (59.3%, n=38/64) were symptomatic. These findings are in contrast to a previous Pakistani study, which reported that almost all the patients with a mucosal anomaly on MRI brain would have at least one symptom [5]. Their study also reported that almost 97% of the anomalies were mucosal thickening. With a larger sample size and a longer duration, our study serves to report a clearer picture regarding the incidence of such anomalies in our population. Our findings are, however, consistent with the studies conducted by Moser et al. [8] and Wani et al. [9], who reported the incidental paranasal sinus anomalies to be 24.7% and 37.5% in their cohorts, respectively. Having said that, the incidence of paranasal sinus anomalies continues to vary across populations as reported by various studies, one example being an Australian study [10], which reported a far lesser (8%) overall incidence of incidental mucosal thickening on MRI brain compared to our cohort. However, it reported the number of symptomatic patients with incidental paranasal sinus anomalies to be 58%, which was consistent with our study.

It is pertinent to mention that the methodology of our study was fairly similar to the majority of the studies in the literature, making the comparison relevant and valid. We hypothesise that the differences in both incidence and symptomology can be explained by the variable geographic factors, such as climate, air quality index, pollution, as well as ethnic differences. Seasonal variation is another important factor that may affect the overall incidence and symptomatology of these incidental findings [8-10]. Also, the fact that our study particularly took into account the incidence of mucosal thickening of <4 mm to ascertain its clinical significance in terms of symptomology may explain the difference. Many studies performed in the past excluded patients with a mucosal thickening of less than 3 mm, which could have missed a certain number of patients in this particular category [10,11]. Our study aimed to fill this gap by reporting their presence with symptomology of the patients.

Our study reported that patients with mucosal thickening of more than 4 mm and those with a mucosal thickening of 2-4 mm were more likely to have related symptoms (p-value of 0.02 and 0.04, respectively) than those with a mucosal thickening of less than 2 mm (p-value=0.08). Similar results were reported by previous studies [8,10,12].

Consistent with the previous studies [13,14], we found that the size of the paranasal polyp is an important determinant of the presence of any symptoms, and reported that patients having small paranasal sinus polyps are likely to be asymptomatic in clinical settings (p-value <0.001). Our study reported a relatively lower frequency of both fluid levels and retention cysts in our cohort; we didn’t find any statistically significant association of these anomalies with the presence of symptomology, just like previously conducted studies [5,11].

According to our study, the most frequently involved sinus with mucosal thickening was the ethmoid sinus (71.8%, n=46, p-value=0.001), followed by the maxillary sinus (65.6%, n=42, p-value=0.003), while the stratification of mucosal thickening by gender found that male patients were more likely to have mucosal thickening in ethmoid sinuses than their female counterparts. Both these findings are in contrast to the findings of a previous study by Hansen et al. [11], which reported a higher incidence of maxillary sinus involvement with more female preponderance. Although their study performed MRI using a 1.5 T HDx scanner (Signa; GE Healthcare Technologies, Inc., Chicago, Illinois, United States), the rest of the study parameters were quite similar to our study. This difference again could be ascribed to the geographic and ethnic differences amongst the respective study populations. Our findings are consistent with a study by Cooke et al., who reported that the maxillary and ethmoid sinuses were the most commonly affected [14].

There were multiple limitations to our study. Despite a bigger sample size compared to previous local studies, our study was performed at a single centre, which limits its generalizability. Our study also could not take into account the various factors, such as seasonal variations, air quality, and environmental pollution, that could affect the incidence and symptomatology of various paranasal sinus anomalies, particularly mucosal thickening. The MRIs were performed using brain protocols optimised for intracranial pathology, not dedicated sinus imaging, which may have affected the detection of subtle abnormalities in the paranasal sinus. We did not offer any interventions to the symptomatic patients with paranasal sinus anomalies to see whether the symptoms reported by the patients were really due to the presence of these incidental findings or not. However, we are currently following the patients in coordination with their respective departments to assess the effectiveness of any medical or surgical interventions that may be offered to the symptomatic patients with incidental paranasal sinus anomalies. Further large-scale prospective studies are needed to ascertain the long-term clinical importance of these incidental paranasal sinus anomalies and the benefit of any interventions being offered to the affected individuals.

## Conclusions

The prevalence of incidental paranasal sinus abnormalities on MRI brain and their symptomatology vary across populations. Both clinicians and radiologists should be aware of their clinico-radiological significance for a better management plan. Patients with mucosal thickening of more than 2 mm are more likely to present with symptoms than other paranasal sinus abnormalities on MRI of the brain. Male patients are more likely to have symptomatic ethmoid sinus mucosal thickening than their female counterparts. At the same time, clinicians should be aware of the benign nature of incidental findings on MRI and may counsel the patients regarding their benign nature and a conservative management plan, to avoid unnecessary anxiety amongst the patients. The MRI forms may be properly filled to provide adequate history, so that the reporting radiologists may form a precise opinion regarding the incidental finding of paranasal sinuses on MRI brain.

## Appendices

Questionnaire for patients reporting for MRI brain to the radiology department, from  various clinics

Section A: Demographic Information

Age (years): ______

Gender: ☐ Male ☐ Female

Known Medical Conditions (if any): ☐ Diabetes Mellitus ☐ Hypertension ☐ Allergic Rhinitis ☐ Asthma ☐ None ☐ Other: ___________

Current Use of Nasal Medications (sprays, drops, antihistamines): ☐ Yes ☐ No

Section B: Paranasal Sinus-Related Symptoms

Have you experienced any of the following symptoms in the past 4 weeks? (Please tick all that apply)

Nasal Symptoms: ☐ Nasal blockage / congestion ☐ Nasal discharge ☐ Post-nasal drip

Facial Symptoms: ☐ Facial pain ☐ Facial heaviness / pressure

Headache: ☐ Yes ☐ No

Olfactory Disturbance: ☐ Reduced sense of smell ☐ Loss of smell ☐ Normal

Allergy-Related Symptoms: ☐ Sneezing ☐ Itchy nose ☐ Watery eyes ☐ None

Severity of Symptoms (overall): ☐ Mild (does not affect daily activities) ☐ Moderate (some limitation of activities) ☐ Severe (significant limitation of activities)

Section C: Symptom Duration

Duration of Symptoms: ☐ Less than 2 weeks ☐ 2-4 weeks ☐ More than 4 weeks

Section D: Clinical Classification (for Research Use)

Based on the above responses, the patient is classified as: ☐ Symptomatic (at least one sinus-related symptom present) ☐ Asymptomatic (no sinus-related symptoms)

Section E: Consent

I confirm that the information provided above is accurate to the best of my knowledge and I consent to the use of my anonymized data for research purposes.

Patient Signature: __________Date___
 

TO BE FILLED BY RESEARCHERS ONLY

A. Type of Paranasal Sinus Anomaly (Tick all that apply)

☐ Sinus mucosal thickening ☐ Retention cyst ☐ Sinonasal polyp ☐ Other (specify): __________________ ☐ No abnormality detected

B. Severity of Mucosal Thickening (Complete only if mucosal thickening is present)

☐ ≤ 2 mm ☐ 2.1 - 4 mm ☐ > 4 mm

C. Sinus Involvement

(Multiple selections allowed)

☐ Maxillary sinus ☐ Ethmoid sinus ☐ Frontal sinus ☐ Sphenoid sinus

D. Laterality

☐ Right ☐ Left ☐ Bilateral

E. Size / Extent of Lesion

☐ Focal ☐ Diffuse ☐ Segmental

F. Specific Anatomical Location

(If applicable)

☐ Sinus floor ☐ Sinus roof ☐ Medial wall ☐ Lateral wall ☐ Ostio-meatal complex ☐ Entire sinus cavity

G. Overall Radiological Classification

☐ Likely incidental finding ☐ Possibly clinically significant ☐ Indeterminate
